# Basic research in HIV vaccinology is hampered by reductionist thinking

**DOI:** 10.3389/fimmu.2012.00194

**Published:** 2012-07-09

**Authors:** Marc H. V. Van Regenmortel

**Affiliations:** Stellenbosch Institute of Advanced Study, Wallenberg Research Center at Stellenbosch University,Stellenbosch, South Africa

**Keywords:** antibody affinity maturation, antibody polyspecificity, discontinuous protein epitopes, HIV vaccines, rational vaccine design, reductionism, reverse vaccinology, systems biology

## Abstract

This review describes the structure-based reverse vaccinology approach aimed at developing vaccine immunogens capable of inducing antibodies that broadly neutralize HIV-1. Some basic principles of protein immunochemistry are reviewed and the implications of the extensive polyspecificity of antibodies for vaccine development are underlined. Although it is natural for investigators to want to know the cause of an effective immunological intervention, the classic notion of causality is shown to have little explanatory value for a system as complex as the immune system, where any observed effect always results from many interactions between a large number of components. Causal explanations are reductive because a single factor is singled out for attention and given undue explanatory weight on its own. Other examples of the negative impact of reductionist thinking on HIV vaccine development are discussed. These include (1) the failure to distinguish between the chemical nature of antigenicity and the biological nature of immunogenicity, (2) the belief that when an HIV-1 epitope is reconstructed by rational design to better fit a neutralizing monoclonal antibody (nMab), this will produce an immunogen able to elicit Abs with the same neutralizing capacity as the Ab used as template for designing the antigen, and (3) the belief that protection against infection can be analyzed at the level of individual molecular interactions although it has meaning only at the level of an entire organism. The numerous unsuccessful strategies that have been used to design HIV-1 vaccine immunogens are described and it is suggested that the convergence of so many negative experimental results justifies the conclusion that reverse vaccinology is unlikely to lead to the development of a preventive HIV-1 vaccine. Immune correlates of protection in vaccines have not yet been identified because this will become feasible only retrospectively once an effective vaccine exists. The finding that extensive antibody affinity maturation is needed to obtain mature anti-HIV-1 Abs endowed with a broad neutralizing capacity explains why antigens designed to fit matured Mabs are not effective vaccine immunogens since these are administered to naive recipients who possess only B-cell receptors corresponding to the germline version of the matured Abs.

## INTRODUCTION

Before it was recognized that the vast majority of epitopes in proteins are discontinuous, i.e., composed of surface residues originating from distant parts of the protein sequence, short peptide segments of viral proteins able to react with antiprotein antibodies were considered to be continuous epitopes mimicking the antigenicity of the protein. This led to the expectation that if such peptides could be made to adopt the 3D structure observed when the corresponding regions of the viral protein are bound to neutralizing monoclonal antibodies (nMabs), the peptides would be able to act as effective vaccine immunogens. More than a thousand peptides were tested over the years as potential synthetic peptide vaccines against a variety of pathogens but not a single peptide passed phase III clinical trials nor was marketed for use in humans (Hans et al., 2006). These attempts to develop synthetic peptide vaccines were based on the premise that continuous epitopes reacting with antibodies specific for a viral protein may be able to induce antibodies that recognized the virus and neutralized its infectivity. It was usually found, however, that few continuous epitopes of viral proteins were able to elicit antibodies that recognized the native protein although most of them readily induced antibodies that reacted with the peptide immunogen (Van Regenmortel and Muller, 1999; Van Regenmortel, 2009a).

In order to be useful as a vaccine immunogen, a peptide must not only induce antipeptide antibodies but must also possess so-called cross-reactive immunogenicity, i.e., the ability to induce antibodies that recognize the cognate protein, as well as cross-protective immunogenicity, i.e., the ability to induce antibodies that neutralize the infectivity of the pathogen (Van Regenmortel, 2006). Since very few linear peptides were found to possess the required cross-reactive and cross-protective immunogenicity, it became generally accepted that the prospects of developing effective synthetic peptide vaccines were very poor. In the present review, it will be argued that many of the impediments that prevented the development of synthetic peptide vaccines in the past are responsible for the lack of success observed in current attempts to design HIV-1 vaccines based on the 3D structure of more complex discontinuous epitopes.

Arguments will be presented that the failure to develop an HIV-1 vaccine by rational design is mainly due to the underlying reductionist thinking that pervades much basic research in immunology and vaccinology. A reductionist mindset obscures the fundamental divide between antigenicity and immunogenicity, i.e., between the chemical nature of antigen–antibody recognition processes and the biological nature of the immunogenic processes that allow a viral antigen to give rise to a protective immune response in a competent host.

## ANTIBODY POLYSPECIFICITY AND THE RELATIONAL NATURE OF EPITOPES AND PARATOPES

The regions of antigen molecules recognized by antibodies are called epitopes while the regions of antibodies that bind to epitopes are called paratopes. Both regions are usually identified by solving the 3D structure of antigen–antibody complexes and determining which amino acids in the two partners make contact with each other (Sundberg and Mariuzza, 2002). When a paratope is defined solely in terms of residues that make contact with an epitope, it is difficult to account for the observation that the binding activity of an antibody often depends on structural features distant from the paratope itself (Schildbach et al., 1993; Chatellier et al., 1996). Similarly, residues in the antigen that are not in contact with the antibody may be able to affect the binding process, for instance by influencing the conformation or stability of the free form of the protein or by participating in long-range allosteric effects. Complex formation is driven by the free energy change associated with the binding process, and residues away from the contact regions are often able to contribute to this free energy change as demonstrated by the changes in affinity and specificity that result when these residues are mutated (Greenspan and Di Cera, 1999). Mutational studies have also shown that not all contact residues contribute to the interaction energy, confirming that epitopes defined structurally differ from epitopes identified in functional assays (Cunningham and Wells, 1993). Furthermore, the structures visualized in antigen–antibody complexes may differ from the structures of the binding sites in the free molecules, before the processes of mutual adaptation and induced fit that often occur when the two partners interact (Wilson and Stanfield, 1994; Bosshard, 2001; Kim et al., 2011). As a result the structure of an epitope bound to an nMab may not correspond to the structure that is recognized by B-cell receptors (BCRs) during the immunization process and is presumed to be required in a vaccine. It is also known that residues in the antigen that are not in contact with paratope residues may be able to modulate the immunogenic activity of epitopes (Moudgil et al., 1998). It was also found that the sensitivity to neutralization of HIV-1 strains that harbor the core epitope recognized by nMab 4E10 was modulated by amino acid substitutions elsewhere in the viral envelope (Gray et al., 2008). All these observations are consistent with the view that epitopes and paratopes are fuzzy binding sites devoid of clear-cut structural boundaries (Van Regenmortel, 1998a).

Specificity has been defined by Medawar and Medawar (1978) as the complementary relationship existing between an agent and something acted on, which arises from the stereochemical complementarity found between such partners as antigen and antibody, enzyme and substrate, or receptor and ligand. In addition to being defined as a measure of goodness of fit between paratope and epitope, antibody specificity can also be viewed as resulting from the capacity of an antibody to discriminate between two or more antigens (Day, 1990, p. 291; Frank, 2002, p. 42).

Antibodies recognize complementary antigens through the extremely versatile binding sites of immunoglobulin (Ig) molecules which are able to recognize virtually every molecular structure (Nezlin, 1994; Wucherpfennig et al., 2007). The Ig binding site consists of 50–70 hypervariable residues distributed over the six complementarity determining regions (CDRs) of the variable domains of the heavy and light Ig chains. Each Ig binding site contains numerous overlapping and non-overlapping paratope subsites of 10–20 residues, each approximately 2800 A^2^ in area, that are able to bind to different antigens. The surface of one paratope corresponds to only 20–35% of the total surface encompassed by the CDRs of an Ig molecule (Denisova et al., 2010). An Ig molecule may sometimes harbor two non-overlapping paratopes, which will allow it to bind simultaneously to two small antigens (Richards et al., 1975; Bhattacharjee and Glaudemans, 1978; Eisen and Chakraborty, 2010). A more common situation is that paratope subsites present in an Ig molecule at least partly overlap which prevents two different antigens from binding simultaneously to the same Ig. The presence of multiple paratopes in Ig molecules means that antibody molecules are always polyspecific (Frank, 2002).

The polyspecificity of antibodies is also demonstrated by their ability to bind large numbers of small peptides possessing limited sequence similarity and by the fact that many residues of an epitope can be replaced by any other amino acid without impairing the epitope’s antigenic reactivity (Getzoff et al., 1988; Geysen et al., 1988). When peptide libraries are tested for their ability to bind Mabs raised against a protein, it is usually found that many peptides that bind Ig residues situated outside the paratope region show little sequence similarity with the target antigen. Such peptides are therefore poor mimics of any epitope of the native protein although they are often referred to as mimotopes (Van Regenmortel, 2009a; Denisova et al., 2010; Irving et al., 2001, 2010).

The fact that every antibody molecule always harbors numerous paratopes, allowing it to bind many related or unrelated epitopes, was recognized as soon as myeloma proteins and Mabs became available and their specificity could be analyzed (for a review, see Eisen and Chakraborty, 2010). It is therefore astonishing that the implications of antibody polyspecificity for vaccine development were pointed out only recently (Van Regenmortel, 2011a, 2012) since the degeneracy of the immune system has been known for many years (Sperling et al., 1983; Parnes, 2004; Wucherpfennig et al., 2007).

The entire surface of proteins harbors a large number of overlapping discontinuous epitopes which can be identified only when many Mabs reacting with the protein have been isolated. Discontinuous epitopes consist of two to five short linear stretches of residues that are distant in the protein sequence but are brought together by the folding of the peptide chain. Since the number of Mabs available for each protein is usually limited, it may seem that antigenicity is located in discrete epitope regions rather than forming an antigenic continuum at the surface of the protein (Van Regenmortel, 2009b). Since discontinuous epitopes cannot be isolated in active form from the protein in which they are embedded, it is impossible to study their capacity to act as effective vaccine immunogens on their own. The intact, native protein must always be used as immunogen and this inevitably produces a heterogeneous response against the numerous epitopes present in the protein (Van Regenmortel, 2012).

A second type of protein epitope called a continuous epitope is defined as any linear peptide region of the protein, usually 5–8 residues long, that is able to cross-react with antibodies raised against the protein. Continuous epitopes usually do not exist as individual, discrete binding or immunogenic sites in the native protein, and only some of their residues may be present at the surface of the protein where they are usually part of discontinuous epitopes (Chen et al., 2009).

**FIGURE 1:**
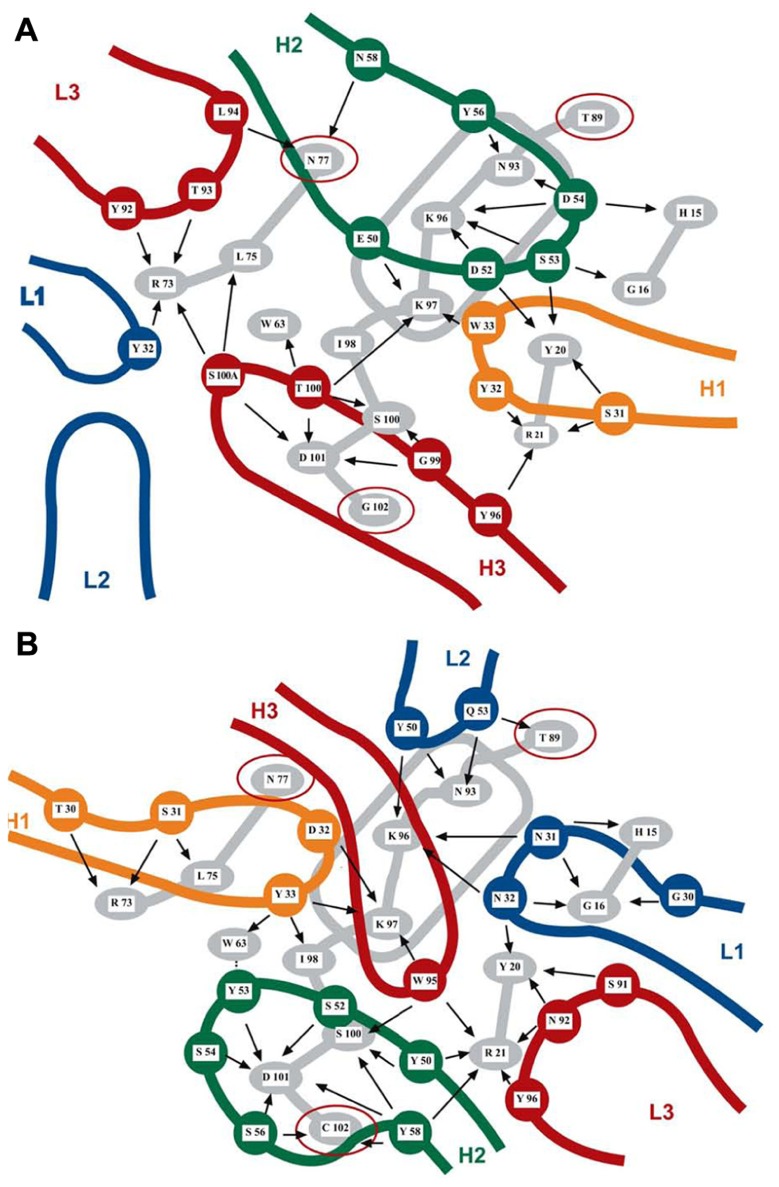
Two overlapping discontinuous epitopes of lysozyme recognized by Mabs F9-13.7 **(A)** and HyHEL 10 **(B)** elucidated by X-ray crystallography. Thirteen residues of lysozyme (in gray) are recognized by both antibodies, albeit with different bonding patterns. The rounded rectangle in gray represents the lysozyme α-helix. The two sets of CDRs are shown in color and have different orientations on the lysozyme surface. Three residues (N77, T89, and G102 highlighted with red circles) are not shared by the two epitopes. Intermolecular contacts are shown by arrows. Mab HyHEL 10 forms a salt bridge between lysozyme K97 and residue D32 of the H1 antibody loop. Mab F9-13.7 forms salt bridges between lysozyme residues K97, K96, and H15 and respectively residues E50, D52, and D54 of the H2 antibody loop (adapted from Lescar et al., 1995, reproduced with permission).

The same residues at the surface of a protein may contribute to several overlapping discontinuous epitopes recognized by different antibodies that present no similarity in their CDRs. As illustrated in **Figure 1** for two lysozyme antibodies, two Mabs recognizing what appears to be a very similar epitope may bind through completely different paratopes that show no similarity in chemical bonding pattern. Epitopes and paratopes are in fact relational entities that can be defined only by their mutual complementarity since they depend on each other to acquire a recognizable identity. Epitopes and paratopes are thus not intrinsic structural features of antigens and antibodies, respectively, because they do not exist as specific sites in the absence of a complementarity nexus between two partners (Van Regenmortel, 2009b). This means, for instance, that the two paratopes illustrated in **Figure 1** do not actually recognize the same epitope. In view of the relational nature of epitopes, the number of epitopes in a protein is equal to the number of different Mabs that can be raised against it. This number was found to be 115 for the insulin molecule (Schroer et al., 1983) and more than a thousand for the BLyS molecule (Edwards et al., 1993). Another consequence of this relational nexus is that analyzing the antigenicity of a protein is equivalent to analyzing the size of the immunological repertoire of the host immunized with the protein.

Antibodies are often given names that suggest they are specific for particular proteins or antigenic sites which always harbor numerous epitopes. This practice can lead to confusion since an antibody cannot be specific for a multiepitopic antigen as a whole but only for one of its epitopes. There is therefore no clear answer to the question whether an Mab or a polyclonal antiserum is the more specific reagent since it depends on which antigens the investigator is trying to differentiate (Van Regenmortel, 1998a). An individual Mab is usually better able to discriminate between two cross-reactive epitopes than a polyclonal antiserum, although the antiserum tends to have greater specificity for a multiepitopic protein. The reason for this is that the many antibodies in an antiserum which recognize the same protein through separate epitopes give rise to an additive specificity effect. Since each polyspecific Ab in the polyclonal serum cross-reacts with a large number of epitopes that are different for each Ab, the collective cross-reactive potential of the antiserum is diluted out which leads to the separate cross-reactivities of the different antibodies being masked (Talmage, 1959; Richards et al., 1975; Al Moudallal et al., 1982; Parnes, 2004). The ability of the immune system to specifically recognize a huge number of multiepitopic antigens is therefore not due to the existence of myriads of antibodies, each one recognizing a unique epitope present in only one antigen, but arises from the combinatorial effect of several polyspecific antibodies recognizing separate epitopes on the same antigen (Wucherpfennig et al., 2007; Van Regenmortel, 2012).

## CAUSATION AND EXPLANATION IN BIOLOGY AND IMMUNOLOGY

Philosophers usually describe the process of scientific explanation by the so-called deductive-nomological model which presents explanations as being logically deduced from one or other relevant law of nature (Klee, 1997; Psillos, 2002). For instance, the occurrence of an eclipse will be explained using Newton’s laws of universal gravitation together with certain initial conditions. Newton’s laws of motion apply to celestial bodies because they move in an interstellar vacuum, free from external interference. On Earth, the laws of Newtonian mechanics explain phenomena only if one adds a *ceteris paribus* (i.e., other things being equal) clause to rule out interfering forces such as electromagnetic forces (Cartwright, 1983). Newton’s laws have sometimes been blamed for leading scientists to expect that all explanations in science should be able to provide the precise predictions achievable in astronomy. In physics, there are many well-established laws that do lead to extremely reliable predictions and have given us a remarkable understanding of the physical universe. Unfortunately, this is not possible in biology for the simple reason that there are no universal laws in biology (Dupré, 1993). Causal explanations in terms of a single cause acting according to a law of nature are therefore not possible in the biological sciences.

Causation is a mode of event generation (Bunge, 2003) and causal relations are relations between successive events and not between two material objects or between a structure and an event. A biological event such as the binding reaction between an antibody and an antigen is thus not caused by the structure of the reactants. An antibody on its own possesses no causal efficacy in bringing about a biological activity such as infectivity neutralization which involves a ternary relationship between antibody, pathogen, and host and always depends on numerous immunological and pathophysiological factors.

In biology, only contributory causes can be identified because a multiplicity of background conditions or factors are always involved in bringing about an effect (Van Regenmortel, 2002a). Because of synergy and various interference phenomena, there is also no linear relationship between the magnitude of one causal factor and the magnitude of a biological effect. Since any observed effect always results from the complex network of interactions and internal regulations that exist in every biological system, a single causal factor can never be presented as an explanation since it is not realistic to assume that the clause “other things being equal” is relevant when hundreds of background conditions contribute to an effect. In non-linear dynamic systems, the notion of causality has very little explanatory value (Berger, 1998; Wagner, 1999).

The immunogenicity of an epitope is a biological property, partly determined by its intrinsic chemical structure, which mainly depends on numerous extrinsic factors such as the host Ig repertoire, the presence of appropriate BCRs and T cell help, the use of adjuvants, the process of antibody affinity maturation, self-tolerance, and various cellular and regulatory mechanisms that exist only in the biological context of the immunized host. Explaining immunogenicity by referring to its multiple “causes” is therefore not helpful for controlling a process that is always influenced by a large number of interdependent and cooperative biological interactions.

**FIGURE 2:**
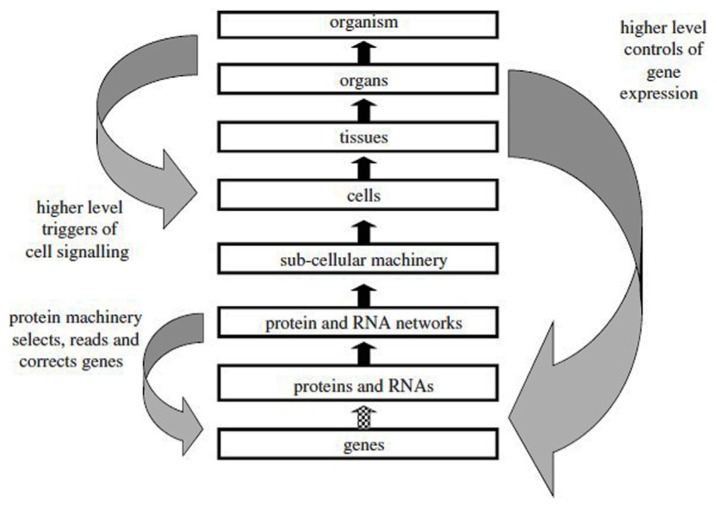
** The reductionist upward causal chain from genes to organisms, and various forms of downward causation that regulates lower level components in biological systems.** This representation does not take into account the influence of the environment at the various levels (from Noble, 2012, with permission).

The dictum “*structure determines function*” has many adepts among molecular biologists and often leads to the assumption that there is a linear causal pathway operating in biomolecules which links gene sequence to protein sequence and then to protein conformation, binding, and function. Such a pseudo-causal pathway appears to possess explanatory power because it is presented in a context-independent manner as if the chemical and cellular environment did not contribute to the process of protein folding or that the natural Darwinian selection process that led to the existence of a functional binding partner could be ignored (Van Regenmortel, 2002b). Such a hypothesized linear pathway is often presented as shown in **Figure 2**, which suggests that the unidirectional flow of genetic information that occurs from DNA to protein can be extended to the upper levels of biological complexity present in tissues, organs, and organisms. However, genes do not cause phenotypic characters and although they contribute to determining phenotypic traits, they do not act upon them. The prior state of a thing is also not the cause of its subsequent state, the caterpillar not being the cause of the butterfly (Mahner and Bunge, 1997, p. 39). Although it has been said that to explain an event is to provide some information about its causal history (Psillos, 2002, p. 217), this does not mean that it is possible in immunology to demonstrate a single chain of successive causes and effects where each effect is itself the cause of a subsequent effect. The arrows pointing upwards in **Figure 2** should thus not be interpreted as representing a single causal relation linking events occurring at different levels and providing an ultimate explanation of an immunological mechanism in terms of the genome. In the case of antibodies, Ig genes are randomly spliced together from gene segments and they undergo extensive somatic hypermutation following activation with antigen; antibody specificity is therefore determined by genetic mechanisms that are unique to the immune system.

There is no single upward causality chain linking all the levels of biological organization since the phenotypes observed at each level result from innumerable interactions between genetic, epigenetic, biochemical, and environmental factors. Although the misleading metaphors of codes, instructions, blueprints, and genetic programs continue to be used to describe the transmission of information from genes to proteins and phenotypes, the misconceptions that arose from the use of such terms are now generally recognized (Kay, 2000; Noble, 2008; Shapiro, 2009). The correspondence between a particular codon and the amino acid it codes for is usually described as being arbitrary or symbolic (Maynard-Smith, 2000). However, it is equally plausible that the code is not arbitrary but originated from the need to have a pattern of hydropathic complementarity between the peptides specified by the sense and antisense strands of DNA molecules (Blalok, 1990; Tropsha et al., 1992). It is, indeed, remarkable that there is no exception to the rule that codons and anticodons always code for amino acids of opposite hydropathicity, i.e., either hydrophilic or hydrophobic residues. This leads to peptides coded by the two DNA strands that tend to bind to each other. This phenomenon explains why peptide analogs that retain the original hydropathic profile found in a continuous epitope are usually able to bind the same antibody although they possess little or no sequence similarity with the original epitope (Hanin et al., 1997; Van Regenmortel, 1998b).

The earlier confusion between the concepts of gene as a predictor of phenotypic characters and a developmental factor in ontogeny has been resolved (Bradie, 2003; Moss, 2003) and it is now evident that our considerable understanding of the mechanisms that allow genes to be translated into proteins has in fact given us very little insight on how phenotypic traits are produced in organisms (Oyama et al., 2001; Oyama, 2009). Although a gene-centric approach to understanding biological systems is still sometimes advocated (Brenner, 2010) it is generally accepted that there is no privileged level of determination in biology at any of the levels illustrated in **Figure 2** and that both bottom up and top down determination can occur (Mahner and Bunge, 1997; Noble, 2012). Since the hierarchy of levels labeled “up” and “down” are actually metaphors (Noble, 2012), one can also refer to horizontal causation for describing the internal causal relations that operate within the boundaries of each of the different levels.

Any level in the hierarchy of biological complexity can be the starting point for a causal analysis, provided a certain initial state of affairs is considered to be in need of an explanation. However, the explanations must be framed in terms of network causality and they always have to consider the numerous factors that simultaneously influence the system in a given biological context. The upward and downward arrows in **Figure 2** therefore represent only one factor among many others that contribute to the observed effect. In all cases, a causal link attributed to one particular factor or mechanism will have to be confirmed by subsequent experimentation which alters that factor since this is the only way to establish that the hypothetical cause elicits the predicted effect in a particular context (Van Regenmortel, 2007). This means that in immunology, explanations in terms of causal links must be validated by experimental interventions and manipulations in order to satisfy Woodword’s (2003) requirement of “making things happen” (see Scientific Understanding Arises from the Ability to Successfully Manipulate the Immune System).

## REDUCTIONISM AND EMERGENCE IN IMMUNOLOGY

The reductionist mindset is epitomized by the assertion of Crick (1966): “The ultimate aim of the modern movement in biology is to explain all biology in terms of physics and chemistry”. Most biologists believe that organisms are composed solely of atoms and molecules without the participation of extraneous or spiritual forces, and they tend to take the view that biological systems can therefore be fully described and understood in terms of the physico-chemical properties of their constituent parts. In viral immunology, reductionist thinking leads to the expectation that both cell-mediated immunity (Zajac and Harrington, 2008) and antibody-mediated immunity to viruses (Neurath, 2008) will eventually be fully understood in terms of the molecular properties of T-cell receptors (TCRs), BCRs, antibodies, MHC molecules, cytokines, and various other cellular constituents.

It is true that methodological reductionism which dissects a biological system into its constituent parts has been extremely successful as a research strategy in molecular biology and immunology and this seems to accredit the view that biology is reducible to physics and chemistry. It is, therefore, somewhat paradoxical that in recent years biologists have become increasingly dissatisfied with the view that biological systems can be fully explained in a reductionist manner using physico-chemical principles. Misgivings about the validity of reductionist explanations in biology have been expressed at several international meetings (Bock and Goode, 1998; Van Regenmortel and Hull, 2002; Byerly, 2003; Kistler, 2003; Van Regenmortel, 2004a) and the claims of genetic reductionism which link human traits to genes have become totally discredited.

Reductionists assume that the behavior of a biological system can be understood by analyzing the multiple interactions that exist between its constituents and that the activity of the whole can be inferred, deduced, calculated, and predicted from the properties of the parts. Antireductionists disagree and claim that this is not feasible because interconnections and interactions between the parts as well as certain inputs from the environment give rise to novel, emergent properties which are absent in the parts taken in isolation and cannot be predicted or deduced from the properties of the parts (Holland, 1994). Examples of emergent properties are the viscosity of water (individual water molecules have no viscosity), a melody arising from notes and the immunogenicity of a protein. Since dissecting the immune system into its components severs the connections that link the various parts together in a functionally integrated manner, essential features that regulate the system’s behavior are destroyed and it is no longer possible to account for the workings of the system as a whole (Van Regenmortel, 2002a). Negative feedback and feed-forward control lead to dynamic behavior that cannot be predicted satisfactorily by linear mathematical models that disregard cooperativity, synergy, and non-additive effects. Emergent properties have their own distinctive causal powers exemplified by the downward causation that occurs when an organism controls biochemical activities that take place at a lower level.

Proponents of reductionism often claim that explanations of biological phenomena can be provided without invoking biological concepts at all. This leads to “Nothing but-ism” statements of the type: “Organisms are nothing but physico-chemical systems” and “the mind is nothing but neural activity” (Van Regenmortel, 2004a). A similar confusion arises when it is claimed that a biochemical process such as the interaction between actin and myosin causes the physiological process of muscle contraction. A frog will jump because it sees a snake and not because a biochemical reaction causes a physiological activity. The biochemical and physiological processes occur simultaneously and the one cannot cause the other since both are descriptions at different levels of the same reaction (Rose, 1998). When the interaction between a viral epitope and a paratope is described at the molecular level, it is debatable whether the analysis belongs to the biological field of viral immunology or to the chemical field of protein science. Although such an analysis could be presented as a reduction of biology to chemistry, it can also be interpreted as an analytical shift that no longer addresses a biological question, especially if no attempt is made to study the interaction as a biological recognition phenomenon between pathogen and antibody or as part of an infectivity neutralization process (Van Regenmortel, 2002b).

Biochemists and structuralists tend to regard the function of a protein simply as what the molecule does, i.e., its functioning or activity at the molecular level. However, the function of an enzyme or an antibody may become meaningful only when the biological context is taken into account and the study takes place at the cellular or organismic level. The activity of an enzyme such as trypsin can be analyzed at the chemical level in terms of which peptide bonds are cleaved but its biological function may become apparent only when the enzyme participates in protein degradation and digestion processes. Similarly, the antigenic and immunogenic activities of a viral discontinuous epitope may become actualized only if the epitope is studied when embedded in a native protein at the surface of a virion or when it interacts with a BCR during the immunization process. When the activity of an epitope is reduced to a chemical interaction with an antibody, the functional implications of such a recognition event cannot be predicted especially if the binding occurs simultaneously with numerous reactions of other antibodies present in a polyclonal antiserum. Extrapolating from the neutralizing activity observed with one Mab to the collective neutralization potential of an antiserum is always hazardous (Mascola et al., 1997) since the initial binding of an antibody often leads to conformational changes in the antigen that modify its ability to interact with other antibodies.

The structural parameters of an epitope bound to an Mab relate only to its binding activity as an antigen since the epitope structure is unlikely to be identical with the immunogenic structure that initiated the immunization process and gave rise to the Mab (Wilson and Stanfield, 1994; Bosshard, 2001; Kim et al., 2011). Recent studies involving deep sequencing of antibodies in human serum have shown that the initial immunogen which triggers the affinity maturation process leading to a particular nMab usually recognizes a germline-like version of the BCR that differs considerably from the one corresponding to the mature Mab, implying that different epitopes are involved (Xiao et al., 2009).

Questions regarding the origin of the binding activity of antibodies cannot be answered in a mechanistic manner by analyzing their internal structure. The answer as to why antibodies are organized the way they are does not lie inside but outside of the antibody molecule. The answer, therefore, cannot be obtained in a reductionist manner by analyzing the antibody’s chemical composition and structure (Cohen and Stewart, 1994, p. 243). Instead of looking for structural explanations of an observed functional activity, biologists emphasize functional explanations for a currently observed biological structure in terms of natural selection and superior fitness in the past (Rosenberg, 1994, p. 193; Van Regenmortel, 2002a). Using the terminology of Mayr (1961) biologists rely on so-called ultimate, historical causes rather than on proximate causes for explaining the functional activity of biomolecules (Laland et al., 2011).

Predicting the binding and functional activity of a protein *de novo* solely on the basis of its structure remains an impossible task. What is possible is to predict the probable activity of a protein by comparison with a structurally similar protein of known activity. In the case of antibodies, their multiple binding activities depend on the existence of a relational nexus with several unknown and unpredictable partners. Attempts to predict the neutralizing capacity of an antibody from its structure is even more unrealistic since it involves a ternary interaction between antibody, antigen, and host which is entirely context-dependent. Furthermore, it is impossible to instruct the immune system to produce polyclonal antibodies endowed with a synergistic neutralizing capacity, regardless of whether the structural correlates of that activity are known or unknown.

Vaccination as an immunological intervention is meaningful only at the level of an entire organism, since organs, tissues, or molecules cannot be vaccinated. The protection against disease which is the goal of all vaccination procedures therefore remains firmly anchored in the complexities of biological systems and cannot be reduced to the chemical level of a molecular interaction (Van Regenmortel, 2004b).

## HUMAN INTENTIONALITY AND RATIONAL DESIGN STRATEGIES

The term “design” can be defined as the deliberate conceiving of an artificial, novel object or process by an intelligent being. The designer’s task is to pose and solve an inverse problem namely to imagine, using available knowledge, what would bring about a desired outcome (Bunge, 2003). In most cases, possible solutions must be tested by trial-and-error experiments until the preset goal is attained. This means that opposing rational design and empirical approaches in vaccine research (Karlsson-Hedestam et al., 2008) is fallacious since all scientific knowledge in the experimental sciences is derived from empirical testing that is necessarily planned and analyzed in a rational manner. One empirical research area that may justify additional investigations is the use of chemically inactivated HIV-1 immunogens. Several new methods are currently available to chemically inactivate the virus while retaining the functional integrity of the Env protein (Van Regenmortel, 2011a and references therein). It may thus be worthwhile to re-examine the potential value of a killed, genetically modified HIV-1 vaccine, provided the safety of such material has been established.

Doing something by design is synonymous to doing it intentionally, and human intentions tend to be perceived as the cause of all human behavior and actions. This often leads to the anthropomorphic fallacy that the behavior and activities of all living organisms can be understood in terms of intentions and purposes. Since all the individual components of a biological system contribute in an integrated manner to its functioning and survival, living organisms may give the impression of having been designed. However, biologists no longer use psychological notions of intentions, design, and purposes to explain biological functions since it is universally accepted that organisms were fashioned and shaped by the filter and pressure of Darwinian natural selection (Ruse, 2002; Hanke, 2004; Van Regenmortel, 2007).

It is nowadays commonly believed that rational design offers the best prospects for developing new drugs and vaccines (Bramwell and Perrie, 2005; D’Argenio and Wilson, 2010) and that this approach is vastly superior to the empirical screening and trial-and-error strategies used in the past. It has been claimed, for instance, that: “One of the goals of research in biotechnology is to transform the process of developing a drug from a trial-and-error empirical operation into a rational, structure-based process” (Amzel, 1998). This denigration of empiricism contradicts the fact that even if a molecule or a vaccine has been designed following structure-based predictions, it is still necessary to screen and verify its activity in the biological context in which it is to be used. The label “rational” applied to modern vaccine design is similar to the concept of “rational drug design” which describes the structure-based strategy used in drug development (Kuntz, 1992). This strategy relies on knowledge of the 3D structure of a biological target for predicting and designing candidate molecules that will bind with high affinity and selectivity to the target and inhibit its biological activity. This computer-assisted strategy based on structural bioinformatics and molecular docking has been highly successful, for instance, for developing the antiretroviral drugs that are used to inhibit various HIV enzymes (Wlodawer, 2002; De Clercq, 2009; LaFemina, 2009).

In the biochemical and biomedical literature, the term rational is used to refer to any procedure that is based on the common sense decision to focus on elements of the system under study for which molecular information is available (Obeid et al., 1995; Van Regenmortel, 1999). “Rational” is then synonymous with “reasonable” or “intelligent” and does not correspond to the notion of rationality found in the philosophy of science and which is used in statements such as “scientific judgment is guided by rationality” (Newton-Smith, 1981; Giere, 1988). It has been suggested that rationality should no longer be used as the ultimate justification for scientific procedures and decision making and that it may be preferable to replace it by the concept of “bounded rationality” (Gigerenzer and Selten, 2001; Lucas and Roosen, 2010). Bounded rationality recognizes that when scientists have to reach decisions on how to proceed, they operate under three unavoidable constraints linked to (1) the limited information they have, (2) the limited capacity of their minds to process huge amounts of data, and (3) the limited amount of time they have to make a decision. One consequence of these limitations is that it is then no longer feasible for them to achieve the optimization process that in principle is required from a genuinely “rational” design procedure.

There is one additional reason for being skeptical of the claim that the rational design of vaccines is a realistic endeavor. Although it is possible to rationally design an epitope or antigen so that it will have an improved structural complementarity to one particular nMab, this only represents antigen design in the context of a single epitope–paratope pair and it should not be called immunogen design. When authors discuss the rational design of an HIV-1 vaccine (Douek et al., 2006; Walker and Burton, 2010; Nabel et al., 2011), they only refer to studies that improve the degree of complementarity in one epitope–Mab pair and they do not clarify how an improved antigen could actually be “designed” to become an immunogen capable of generating protective antibodies. Presumably this would require an investigation of the numerous factors, extrinsic to epitope–paratope recognition, that originate in the immunized host and control the type of protective immune response that a vaccine immunogen is expected to elicit. Optimizing the binding activity of an antigen by structure-based design is clearly not equivalent to controlling or improving by trial-and-error the numerous biochemical and cellular interactions involved in the generation of a neutralizing immune response. Antigen design is actually masquerading as immunogen design because of the unacknowleged, implicit assumption that antigenic reactivity necessarily entails an immunogenic capacity to produce antibodies similar to the one used as template for designing the antigen. Although some authors acknowledge the difference between antigenicity and immunogenicity, they still tend to adhere to the view that an antigen designed to fit an nMab will also be an efficient vaccine immunogen able to elicit neutralizing and protective antibodies. All the experimental immunization results obtained so far (Van Regenmortel, 2011a) as well as the nature of the biological processes and mechanisms underlying antibody synthesis and maturation speak against such an assumption (Verkoczy et al., 2011; Chen et al., 2012). It is therefore not astonishing that all the studies reporting the successful rational design of a viral antigen have failed to demonstrate that the engineered antigen is also an effective vaccine immunogen (Van Regenmortel, 2012).

## REVERSE VACCINOLOGY AND THE RATIONAL DESIGN OF HIV-1 ANTIGENS

The concept of reverse vaccinology (RV) was introduced in the field of bacterial vaccines by Rappuoli (2001) about 10 years ago. RV does not attempt to develop vaccine candidates by the usual approach of fractionating bacterial extracts and determining which antigens are able to induce a protective immune response but by predicting potential vaccine immunogens using bioinformatics analyses of entire bacterial genomes. *In silico* analysis of the genome provides a list of all the surface-exposed proteins that the pathogen is able to express and these proteins are then produced by high-throughput technologies and tested for their immunoreactivity with patient sera as well as for their ability to induce protective antibodies (Rappuoli and Bagnoli, 2011). This strategy was called “reverse” vaccinology because the investigators operate in a so-called reverse manner, i.e., starting from the genome rather than from the organism, to discover which proteins are potential vaccine immunogens. This genome-based strategy has the advantage that hundreds of bacterial proteins are identified as candidate immunogens even if the bacteria cannot be cultivated.

In virology, RV has a different meaning altogether and refers to the strategy of generating a vaccine from the known crystallographic structure of nMabs bound to viral epitopes. The term “reverse” is used metaphorically in the sense that the investigator is trying to generate a vaccine starting from neutralizing antibodies instead of trying to generate such antibodies by immunization (Burton, 2002; Walker and Burton, 2010). It is not clear, however, which vaccine discovery process is being reversed since the bnMab is simply used as a template to reconstruct its epitope outside the context of the natural antigen, using structure-based design technology. The assumption is then made that the reconstructed antigen designed to fit the bnMab will possess the immunogenic capacity of inducing a polyclonal antibody response with the same neutralizing capacity as the Mab.

Since the RV approaches used for developing bacterial and viral vaccines are completely different it would be preferable to call them genome-based and structure-based RV, respectively (Van Regenmortel, 2011b). Both strategies require trial-and-error experimentation to ascertain which candidate bacterial or viral proteins are able to act as effective vaccine immunogens, and it is therefore misleading to present RV as a rational design procedure that overcomes the empirical nature of all vaccine science (Mascola, 1999; Karlsson-Hedestam et al., 2008). In section “Human Intentionality and Rational Design Strategies”, it was argued that the development and discovery of vaccine immunogens by rational design is actually a misnomer. The ensuing discussion will therefore only consider attempts to rationally design antigens able to bind Abs without making the assumption that what is being designed is also a vaccine immunogen (Sattentau and McMichael, 2010).

**FIGURE 3:**
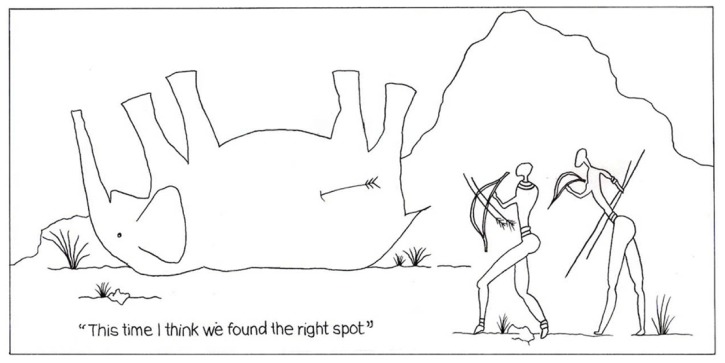
** This cartoon illustrates how one vulnerable epitope region in an elephant is successfully hit by a single nMab arrow**.

In a recent comprehensive review paper entitled: “Structure-based vaccine design in HIV: blind men and the elephant,” Pejchal and Wilson (2010) referred to the Indian tale of three men trying to describe an elephant and reaching different conclusions because they touch different parts of the animal. They compared this unsatisfactory procedure to the way investigators try to identify potential HIV vaccine targets by determining which Env regions are targeted by individual nMabs. An amusing illustration of this approach is shown in **Figure 3** where one vulnerable “epitopic” region in the animal has been hit by a single Mab arrow.

**FIGURE 4:**
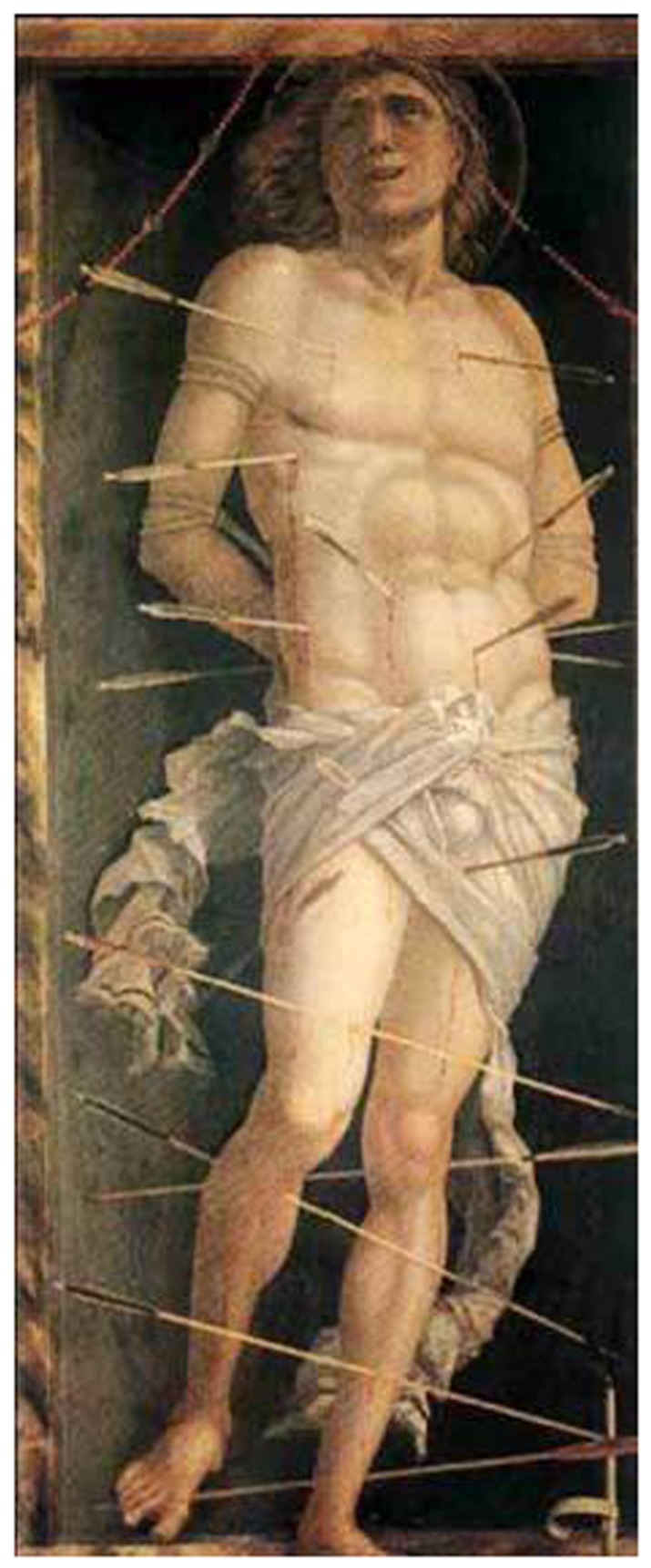
** The painting of St Sebastian by Mantegna in the Ca d’Oro museum in Venice.** The arrows symbolize a polyclonal antibody response which is not directed to a single vaccine target.

The question of whether an effective vaccine can be developed by focusing the immune response on a single vulnerable site or epitope is a controversial issue (Scheid et al., 2009; Walker et al., 2009, 2010; Lynch et al., 2011; Moore et al., 2011). An alternative approach is depicted in the painting of Mantegna (**Figure 4**) which illustrates symbolically that an effective vaccine may require multiple vulnerable regions to be targeted by a polyclonal Ab response (Doria-Rose et al., 2012).

Although the advent of Mabs has revolutionized our understanding of immunochemistry by making it possible for investigators to successfully dissect polyclonal Ab responses in terms of single epitope–paratope interactions, the use of Mabs also had the unfortunate consequence that vaccine hunters have tended to focus on individual epitopes as elicitors of neutralizing Abs. This led them to neglect the synergistic effects that result from the collective neutralizing activities of antibodies directed to different epitopes (Laal et al., 1994; Mascola et al., 1997; Zwick et al., 2001; Van Regenmortel, 2012).

Attempts to design improved HIV-1 antigens have used as templates a small number of nMabs that recognize different antigenic sites of the Env protein such as the conserved CD4-binding site (CD4bs; Burton et al., 1994; Kwong et al., 1998; Zwick et al., 2003; Li et al., 2007), the CD4-induced (CD4i) antigenic site that becomes accessible after gp120 interacts with CD4 (Labrijn et al., 2003), the semiconserved V3 loop (Javaherian et al., 1989; Zolla-Pazner, 2004; Zolla-Pazner and Cardozo, 2010), the membrane-proximal external region (MPER) antigenic site (Zwick, 2005), and the glycan antigenic site (Scanlan et al., 2002; Pantophlet, 2010). It must be emphasized that each antigenic site harbors a large number of different epitopes. This means that if an immune response directed to one HIV-1 antigenic site is considered to represent a single specificity, this does not exclude that a large number of different Abs will recognize overlapping targets within the same antigenic region (Walker et al., 2010).

Various strategies have been used to improve the antigenic reactivity of the epitopes recognized by Mabs by reconstructing the epitopes outside the viral protein context in which they occur. Such strategies include (1) hyperglycosylation (Pantophlet et al., 2003a), (2) amino acid substitutions (Pantophlet et al., 2003b), (3) stabilization of the CD4-bound state by introducing cross-links or deleting variable loops (Dey et al., 2007; Zhou et al., 2007; Wu et al., 2010), and (4) immunofocusing, i.e., decreasing the ability of the CD4bs to bind to non-neutralizing antibodies while retaining the capacity to bind to bnMabs (Pantophlet and Burton, 2003). Although some of the engineered antigenic sites that were obtained reacted better with bnMabs, none of them were found to be effective immunogens able to induce broadly neutralizing antibodies (Pejchal and Wilson, 2010; Kong and Sattentau, 2012; Van Regenmortel, 2012).

Attempts were also made to improve the antigenic reactivity of the MPER continuous epitopes recognized by Mabs 2F5, 4E10 (Muster et al., 1993; Julien et al., 2008) and Z13 (Nelson et al., 2007). This was done by adding flanking residues to the core epitopes (Tian et al., 2002), constraining the epitopes in various conformations (Joyce et al., 2002; Ho et al., 2005), mimicking the conformation of the epitopes when they are bound to bnMabs (Ofek et al., 2004), and grafting the epitopes into various protein scaffolds (Arnold et al., 2009; Burton, 2010; Correia et al., 2010; Ofek et al., 2010a; Guenaga et al., 2011). Some of these modifications increased the binding capacity of the peptides but they did not endow them with the capacity to elicit bnAbs (Law et al., 2007; Guenaga et al., 2011). This may be due to the fact that the short core peptide regions recognized by 2F5, 4E10, and Z13 do not constitute the full epitopes since there is evidence that the epitopes are chemically heterogeneous and include lipid moieties that participate in the binding reaction through hydrophobic interactions (Scherer et al., 2010; Ofek et al., 2010b). Other composite HIV epitopes have been shown to include glycan moieties (McLellan et al., 2011). It has been shown that hydrophobic residues of the long CDR H3 loop of Mab 2F5 are able to insert into the viral membrane, producing major conformational rearrangements in both epitope and paratope and illustrating the role of induced fit dynamics in antigen–antibody interactions (Alam et al., 2009; Kim et al., 2011).

Epitopes in certain HIV-1 strains may become inaccessible to antibodies following hyperglycosylation, mutations or conformational changes, a phenomenon called antigenic masking (Krachmarov et al., 2005). This makes it impossible for such HIV-1 strains to be neutralized by certain nAbs since epitope exposure is usually a prerequisite for neutralization by antibody molecules (for a review, see Pantophlet, 2010). Epitopes in the V3 loop that remain unmasked in many HIV-1 strains and are recognized by bnMabs have been identified using signature motifs of 2–4 residues and it has been suggested that cocktails of such epitopes may be good vaccine candidates (Agarwal et al., 2011). Recently, it was shown that cross-clade nAbs could be induced by using V3-scaffold immunogens in a DNA prime/boost regimen (Zolla-Pazner et al., 2011).

Many attempts have been made to develop vaccine immunogens by expressing surface loops containing continuous epitopes of different viruses as recombinant proteins (Hofnung and Charbit, 1993) but even this fairly straight-forward approach did not produce any effective viral vaccine (Van Regenmortel and Muller, 1999). Compared to simple loop structures, reconstructing HIV-1 discontinuous epitopes (Moore and Ho, 1993; VanCott et al., 1995) and presenting them in the required conformation at the surface of a carrier or scaffold protein is a much more difficult task and all attempts to produce effective HIV-1 vaccine immunogens in this way have so far been unsuccessful (Burton, 2010; Azoitei et al., 2011).

An even more difficult task is to reconstruct epitopes that arise from the quaternary structure of viral proteins. It has been known for more than 40 years that such epitopes which were initially called neotopes (see Van Regenmortel, 2009b, 2012) are present in capsid and membrane proteins and are easily detectable by appropriate immunoassays (Neurath and Rubin, 1971; Van Regenmortel and Neurath, 1985). These epitopes are nowadays commonly referred to as quaternary epitopes which is a somewhat odd terminology since it would correspond to calling continuous and discontinuous epitopes, primary and tertiary epitopes, respectively. Neotopes arise from the juxtaposition of residues in neighboring protein subunits or from conformational changes induced by intersubunit interactions (Van Regenmortel, 2009b). They were shown to be present in HIV-1 gp120 trimers only fairly recently although they appear to be very common (Broder et al., 1994; Cho et al., 2000; Walker et al., 2009; Pantophlet, 2010; Robinson et al., 2010; Zolla-Pazner and Cardozo, 2010; Pancera et al., 2010; Wu et al., 2011a).

Mab 2902 is the first antibody directed to a neotope of the Env protein which had its structure elucidated (Gorny et al., 2005; Kimura et al., 2009). This antibody which is highly strain-specific has a 21-residue long CDR H3 loop which protrudes from the paratope surface and recognizes a neotope comprising portions of the V2 and V3 loops (Honnen et al., 2007; Changela et al., 2011). The two somatically related Mabs, PG9 and PG16 which neutralize about 80% of primary HIV-1 isolates also recognize a V2V3 neotope (Walker et al., 2009; Pejchal et al., 2010; Pancera et al., 2010). The PG9 neotope contains a single β strand and two glycans that form a canyon structure into which the long CDR H3 loop of Mab PG9 inserts itself (McLellan et al., 2011). Many anti-HIV-1 antibodies have been found to possess long CDR H3 regions (Stanfield et al., 2004; Huang et al., 2005; Burke et al., 2009; Changela et al., 2011) although they do not use their protruding loops to reach recessed epitopes in the same way as Mabs PG9 and 2909. Unfortunately, it is not known how antibodies with long CDR H3 loops can be induced by immunization.

Reconstructing HIV-1 neotopes by structure-based design may turn out to be an impossible task, partly because of the unstable and transient conformation of Env trimers (Du et al., 2009) which can alternate between open and closed quaternary conformations (Harris et al., 2011). It remains unclear whether such transient neotopes are advantageous for inducing neutralizing antibodies because their conformational variability is able to facilitate induced fit adjustments and BCR recognition. In studies with other HIV-1 epitopes, there are conflicting reports on whether immunogenicity is enhanced by increasing or decreasing epitope flexibility (Dey et al., 2009; Moseri et al., 2010; Ofek et al., 2010a; Guenaga et al., 2011).

In recent years, large numbers of additional bnMabs have been isolated from HIV-1 infected individuals (Scheid et al., 2009; Simek et al., 2009; Stamatatos et al., 2009; Corti et al., 2010) which demonstrates that the human immune system is able to induce such neutralizing antibodies more frequently than previously thought (Wu et al., 2010; Scheid et al., 2011; Tomaras et al., 2011; Overbaugh and Morris, 2012). This has led to the expectation that additional HIV-1 epitopes and potential vaccine targets will continue to be discovered and that this should facilitate and guide the design of effective vaccine immunogens (Sattentau and McMichael, 2010). Such optimism assumes that the identification of additional HIV-1 epitopes somehow entails that there is then an increased probability that the rational design of HIV-1 vaccine immunogens will become more feasible. It would be equally unwarranted to claim that discovering additional bnMabs useful for passive immunotherapy is likely to improve our ability to determine which immunogens are able to elicit such protective antibodies by active immunization. Although the passive administration of rare human anti-Env bnMabs to non-human primates (NHPs) can protect the animals from virus infection, this type of successful immunotherapy does not in any way tell us how such protective antibodies can be elicited by vaccination. It is of course possible to improve the paratope binding efficacy of a particular Mab intended for passive immunotherapy, using structure-based design. However, as discussed in section “Human Intentionality and Rational Design Strategies”, what is not feasible is to rationally design an immunogen that will elicit a protective polyclonal antibody response of predetermined efficacy (Van Regenmortel, 2012).

## THE BOTTLENECK OF ANTIBODY AFFINITY MATURATION

Considerable knowledge is available regarding the genetic basis of antibody diversity. It has been known for many years that initial exposure to any antigen elicits a primary immune response characterized by low-affinity germline-encoded antibodies (Marks, 1994). In naïve human individuals who have not yet encountered a viral antigen, a primary response is initiated when the antigen binds to germline BCRs derived by random genetic recombination from a small pool of variable (V), diversity (D), and joining (J) gene segments present in the human genome (Arnaout et al., 2011). These BCRs which arise from rearranged heterodimers of light and heavy chains are highly polyspecific and react weakly with a large number of different antigens, allowing the immune system to initiate a response against virtually any antigen. Structural analyses have shown that the polyreactivity of germline Abs is enhanced by the flexibility and conformational versatility of the CDRs (Manivel et al., 2000, 2002; James et al., 2003; Yin et al., 2003; Khan and Salunke, 2012). The polyreactivity of germline Abs and of the initial response to HIV-1 Env antigens is a general property of the human immune system and is not a special feature of HIV immune responses. HIV-1 antibodies have been shown to cross-react with cardiolipin (Haynes et al., 2005a), host cell and gut flora antigens (Liao et al., 2011), as well as with histones, ribonucleoprotein, centromere B, and Ro/Sjogren syndrome autoantigen (Morris et al., 2011). It has been postulated that B cells that produce autoreactive Abs may trigger tolerance mechanisms which could eliminate bnAb precursors (Haynes et al., 2005b).

After initial binding of the antigen to germline BCRs, a stochastic process of somatic hypermutation occurs in the variable regions of Ig genes at rates several logs higher than in other genes. This leads to the selective expansion of B cell clones that possess high affinity BCRs for the immunizing antigen and to the proliferation of plasma cells that secrete antibodies with increased affinity for the antigen.

Advances in large scale pyrosequencing of the antibodyome, i.e., of the complete set of antibody sequences present in an organism at a given time, has allowed deep sequencing of V (D) J gene segments and Ig genes (Fischer, 2011). This made it possible to identify which germline Ig genes are used in a given B cell maturation process and to follow the mutational pathway from naïve, low affinity B cells to high affinity mature BCRs and antibodies (Dimitrov, 2010).

Early studies of antigen-driven maturation in HIV-1 antibodies showed that extensive somatic hypermutation occurred in their variable regions amounting to an average mutation frequency of 10–20% (Andris et al., 1991; Moran et al., 1993; Huang et al., 2004). When the germline predecessors of the affinity-matured HIV-1 Mabs b12, 2G12, and 2F5 were examined, it was found that the antibodies derived from naïve B cells did not bind to the epitopes that were recognized by mature Mabs (Xiao et al., 2009). Subsequent studies revealed that most anti-HIV-1 bnMabs were highly mutated antibodies which had undergone a prolonged affinity maturation process, thereby acquiring a high neutralization potency (Mouquet et al., 2011). The affinity maturation observed in HIV-1 antibodies was much more extensive than the 5–10% mutation frequency usually observed with antibodies directed to other viruses (Zhu et al., 2008; Chen et al., 2012). In Mab VRCO1 which neutralizes about 90% of the HIV-1 strains tested, more than 60 amino acids in the variable region were found to differ from the germline sequence, which amounts to a 30% mutation frequency (Zhou et al., 2010; Scheid et al., 2011; Wu et al., 2011b). In the bnMabs PG9 and PG16, about 20% affinity maturation was present (Pancera et al., 2010) whereas in the less broadly neutralizing Mab2909 the degree of affinity maturation was considerably less. There is now abundant evidence that the degree of affinity maturation in bnMabs correlates with their neutralizing potency.

The germline-like versions of all these Mabs showed little or no measurable binding to HIV-1 Env, indicating that the immunogens which initiated the affinity maturation process are unlikely to have been the epitopes recognized by the mature bnAbs used as templates in the RV experiments. These findings therefore invalidate one of the assumptions underlying attempts to develop an HIV-1 vaccine by rational design (Pantophlet, 2010), namely that the epitopes recognized by matured bnMabs can be used to trigger the affinity maturation process required to obtain protective antibodies.

Some investigators have been unwilling to admit that these findings have led to an impasse in structure-based RV. If somatic hypermutation is indeed a prerequisite for obtaining anti-HIV-1 bnAbs, then it may be mandatory to use vaccine immunogens able to bind germline BCRs rather than immunogens designed to mimic the epitopes recognized by mature bnMabs. It may also be necessary to administer vaccine immunogens over several months or years if large numbers of amino acid changes must accumulate in germline precursor genes to obtain antibodies of sufficiently high affinity and neutralization breadth (Gray et al., 2011; Euler et al., 2012). On the other hand, it is not known if all the mutations observed in matured antibodies are required for achieving an adequate binding and neutralization potency (Burton et al., 2005), nor if the B cells expressing BCRs that bind with low affinity to certain viral antigens will be able to outcompete B cells expressing BCRs that bind to other epitopes with higher affinity.

It has been argued by some that a low affinity interaction between a naïve B cell and an HIV-1 epitope may be sufficient for initiating the affinity maturation process required for developing a protective immune response (for a review, see Van Bubnoff, 2010). However, an even greater hurdle may have to be overcome if it turns out that a stochastic somatic hypermutation process which could follow millions of different pathways (Zhu et al., 2011) and may have to be guided and controlled in every vaccinated individual, is a requirement for developing a protective immune response. This new appreciation of the importance of somatic hypermutation in BCRs has led to the realization that the strategy of using as vaccine immunogens epitopes designed to fit mature bnMabs had little chance of succeeding since the required affinity-matured BCRs do not exist in naïve, vaccinated individuals (Chen et al., 2012).

Attempts are currently being made to try to modify Env molecules so that they become able to bind germline BCRs or maturation intermediates (Wu et al., 2011b). Glycan-depleted Env molecules have been obtained that bind to unmutated precursors of Mab 2F5 and 4E10 but these were unable to elicit neutralizing antibodies (Ma et al., 2011).

## SYSTEMS BIOLOGY AND THE IDENTIFICATION OF IMMUNE CORRELATES OF PROTECTION

Systems biology is sometimes presented as a research strategy that grew out of the realization that studying separately the individual components of a complex biological system would not lead to an understanding of how the whole system works (Cornish-Bowden, 2011). Since biological systems possess emergent, functional properties that are absent in their constituents parts and are characterized by dynamic networks, regulatory mechanisms, robustness, and modularity, it seemed impossible to explain their integrated behavior by considering only events occurring at the level of individual macromolecules and cells (Van Regenmortel, 2004b; O’Malley and Dupré, 2005; Mazzocchi, 2008).

Systems biology is an attempt to describe the multiple interactions between all the parts of a biological system by focusing on the dynamics of the entire system (Ideker et al., 2001; Kitano, 2002). Since the immune system utilizes the dynamic interaction of a wide array of biomolecules, cells, and tissues, the resulting complexity makes the study of immune responses particularly apt to a systems biology approach (Aderem and Smith, 2004). Genomics, proteomics, and other “omics” technologies based on multiplex microarrays, high-throughput sequencing, and bioinformatics are the methods used for this purpose which have made it possible, for instance, to identify candidate antigens for diagnostic, therapeutic, and vaccine applications (Van Regenmortel, 2007). Systems biology is sometimes criticized for being a data-driven approach that cannot lead to the scientific discoveries that follow from hypothesis-driven research (Allen, 2001; Wilkins, 2001) although some authors argue that both approaches are complementary (Kell and Oliver, 2004). It has been suggested that because living organisms have evolved, in response to changing environments, through the accumulation of random, error-prone changes, they developed a type of complexity different from the complex law-like behavior of their underlying physico-chemical constituents (Kelley and Scott, 2008). Biological science therefore does not exhibit the regularities found in the physical sciences and biologists are only able to explain phenomena by positing mechanisms and making predictions without actually offering explanations of how the mechanisms under investigation function. This has led many biologists to adhere to a viewpoint called “epistemological antireductionism” (Nagel, 1998) and to opt for some form of holism, emergentism (Gatherer, 2010), or relational biology (Rosen, 1991; Cornish-Bowden, 2006), while at the same time accepting that many biological phenomena are too complex to be comprehended by human intelligence (Gannon, 2007).

In vaccinology, systems biology approaches have been used mainly for predicting vaccine-induced immunity (Pulendran et al., 2010; Oberg et al., 2011). A proof-of-concept study was done by identifying patterns of upregulated gene expression, called molecular signatures, that were induced in humans vaccinated with the yellow fever vaccine YF-17D (Querec et al., 2009). This vaccine is one of the most successful vaccines ever developed and it was possible to correlate parameters such as magnitude of antigen-specific CD8^+^ T cell responses and several molecular signatures with the development of protective immunity against yellow fever. A major shortcoming of such studies is that it is very difficult to establish if any correlations revealed by genomics or proteomics analyses are causally linked to protection. Genomics analyses may reveal, for instance, that following an immunization protocol, hundreds of genes are up- or downregulated. However, since several gene products always act in combination to generate biological functions, the resulting functional diversity is truly staggering, leading to numbers of potential combinations of causal factors that are larger than the total number of atoms (10^80^) in the Universe (Feytmans et al., 2005; Noble, 2006).

It should also be evident that the presence of immune correlates of protection in vaccines can only be identified retrospectively after an effective vaccine has been developed empirically. In individuals who have survived a natural infection and are protected against reinfection, it could also be feasible to look for immune correlates of protection. However, this is not possible in the case of HIV infection since there are no individuals who have cleared infection and are subsequently immune to reinfection. Studies with long-term non-progressors and elite controllers of HIV-1 infection (Okulicz, 2012) are also of little value since it is not possible with such individuals to exclude an innate or genetic predisposition to non-infection nor to predict which effector functions would be mediated by an adaptive vaccine-induced immunity (Koup et al., 2011). Since it is also impossible to determine immune correlates of protection from failed HIV-1 vaccine trials, it is not astonishing that past attempts to identify such correlates have been unsuccessful. The recent RV144 vaccine trial which showed a modest level of efficacy (Rerks-Ngarm et al., 2009) is the first instance when it became possible to test if immunological assays could serve as surrogate endpoints for HIV-1 infection (Nakaya and Pulendran, 2012; Rolland and Gilbert, 2012). However, the RV144 trial was not designed to identify immune correlates of protection and a retrospective analysis of the small number of samples available from this low efficacy trial was unlikely to reveal which aspects of the antibody response correlated with immune protection (Koup et al., 2011). Only the presence of IgG antibodies to the V1/V2 loops was found to show some correlation with a limited protection against HIV infection in the first year of the trial.

The use of NHP models of HIV infection such as simian immunodeficiency virus (SIV) and simian-HIV virus (SHIV) for evaluating immune correlates is also unsatisfactory because of the differences in disease pathogenesis and immune responses in HIV, SIV, and SHIV infections (Koup et al., 2011). It is also no longer generally accepted that showing protection in NHPs should be a gatekeeper for advancing a particular vaccine product into human efficacy trials (Shedlock et al., 2009; Thomas, 2009; Greek, 2012). A particular product that works in macaques may not work in humans and a strategy that shows no efficacy in NHPs could nevertheless work in humans. Animal models are poor predictors for human responses partly because results depend on the viral strains and doses used for challenge experiments and on the routes of infection (Thomas, 2009). Although it may theoretically be justified to pursue exploratory small-scale human trials when no evidence is available to show that the approach being tested works in NHPs, it is recommended that NHP studies should always be used in the preclinical evaluation of HIV-1 vaccines (Morgan et al., 2008).

## SCIENTIFIC UNDERSTANDING ARISES FROM THE ABILITY TO SUCCESSFULLY MANIPULATE THE IMMUNE SYSTEM

In recent years, many authors have stressed the need to embark on large scale basic research programmes in order to increase our understanding of the human immune system and of HIV-1 antigenic structure and pathogenicity (Thomas, 2009; McElrath and Haynes, 2010; Virgin and Walker, 2010). Such recommendations arise from the realization that our current scientific knowledge is too limited to enable us to instruct the human immune system to generate a protective response against HIV-1 infection. It is not clear, however, whether an increase in our basic knowledge of immunology and of the way bnAbs develop in certain HIV-1 infected individuals will enable us to elicit such antibodies by vaccination.

Instead of emphasizing the importance of theories and basic knowledge for achieving scientific understanding and solving problems in applied science, Hacking (1983) in his influential book *Representing and Intervening*, suggested that it is the ability of experimentalists to successfully manipulate a given system that gives them the confidence that their scientific constructs and entities are real and that a particular scientific explanation is adequate. Whereas observation alone may not justify a belief in the reality of an invisible fuzzy entity such as an epitope, it is our ability to successfully manipulate it in an experiment that convinces us that the entity exists. In other words, we need to interfere with the material world in order to obtain knowledge about it and our scientific understanding increases because we are able to intervene in a system and successfully manipulate the phenomenon under investigation (Kelley and Scott, 2008). This means that we “understand” the immune system when we can manipulate and control it and are able, for instance, to achieve protective immunity by vaccination (Kumar et al., 2012). An understanding of the immune system is thus achieved because of a prior successful intervention. This is different from the usual assumption that we need to increase our understanding of basic immunology in order to be able to manipulate and control the immune system.

In his book *Making Things Happen*, Woodword (2003, p. 9) emphasized that causal relations and explanations are important to human beings because of their interest in manipulating and controlling nature. Although the complexity of the immune system may prevent us from identifying all its internal regulatory mechanisms, it is by trial-and-error experimentation that we discover if the system can be successfully manipulated to achieve protective immunity. Unfortunately, there is no guarantee that increasing our knowledge of viral immunology and pathogenicity will necessarily give us that capacity.

Philosophers of science have used the so-called convergence argument to explain why scientists are confident that their experimental findings justify a belief in the reality of the entities and phenomena they study (Klee, 1997, p. 212). If a variety of experimental results all converge on establishing what is the cause of AIDS, then it would be perverse to follow AIDS denialists and doubt that HIV causes AIDS. Conversely, it seems odd to believe that the failure of hundreds of attempts to transform HIV-1 epitopes recognized by bnMabs into effective vaccine immunogens is simply a remarkable coincidence which does not demand that we modify our underlying assumptions. It certainly is more realistic to accept that the convergence of so many negative experimental outcomes justifies the conclusion that the RV approach that has been used is not appropriate for developing a preventive HIV-1 vaccine.

## CONCLUDING REMARKS

Vaccinologists are well aware that vaccine research is an empirical science (Mascola et al., 1997) and that effective viral vaccines have always been discovered by trial-and-error immunization trials rather than by rational design using the 3D structure of viral antigens. The reasons why successful strategies used in the past failed in the case of HIV are well documented (Hilleman, 1992; Kusters and Almond, 2010; Virgin and Walker, 2010; Kong and Sattentau, 2012; Van Regenmortel, 2012). (1) The natural immune response in HIV-1 infected individuals does not clear the infection and there is therefore no natural immunological mechanism that a vaccine could mimic; (2) during HIV-1 infection, antibodies are mostly elicited against variable and accessible Env loops rather than against functionally important but less accessible conserved domains such as the receptor and co-receptor binding sites; (3) HIV-1 integrates into the host genome and establishes a latent pool of infected cells which conceal the virus from immune recognition; (4) the virus progressively destroys the immune system; (5) HIV-1 isolates exhibit an enormous antigenic variability; (6) the immune system does not readily elicit bnAbs against cryptic and transient HIV-1 epitopes; (7) the degree of antibody affinity maturation required to obtain antibodies that neutralize HIV-1 is much higher than what is needed in the case of antibodies directed to other viruses.

The present review discusses why the reductionist nature of structure-based RV is unlikely to lead to an effective HIV vaccine. Reductionist thinking has been prevalent in molecular biology for half a century and still has a strong hold on investigators who aim to develop a preventive HIV-1 vaccine. The reductionist mindset, for instance, leads them to accept that the biological activities and functions of Abs can be reduced to their physico-chemical structures and that the immunogenic potential of a protein molecule can be deduced from its antigenic properties. Chemical antigenicity is thereby confused with biological immunogenicity. This leads reductionists to assume that it should be possible to control an immune system and have it produce neutralizing antibodies simply by vaccinating an individual with a viral epitope that has been engineered to fit an nMab using structure-based design technology. This assumption overlooks the fact that every anti-HIV-1 bnMab is polyspecific and can bind viral epitopes different from the one identified when the structure of the bnMab–HIV complex was solved. There is therefore no reason why the particular HIV-1 epitope identified by crystallography should be the one that triggered the immune response that gave rise to the Mab.

Since the surface of a viral antigen is an antigenic continuum, the dissection of antigens using individual Mabs tends to give a biased view of immunological specificity and protection against infection since these properties usually result from the collective activities of several different antibodies reacting with the same antigen molecule. By focusing rational vaccine design on single epitope–paratope pairs, the neutralization synergy that tends to occur with polyclonal antibody responses will be missed.

The structural parameters of effective HIV vaccine immunogens have not been elucidated and it is therefore unfortunate that an empirical approach to vaccine development is often denigrated since trial-and-error experimentation remains the best strategy for developing any vaccine.

## Conflict of Interest Statement

The authors declare that the research was conducted in the absence of any commercial or financial relationships that could be construed as a potential conflict of interest.
